# Systematic Review on Mortality in the Elderly on Methadone Maintenance Treatment

**DOI:** 10.7759/cureus.68910

**Published:** 2024-09-07

**Authors:** Farhana Nazmin, Tuheen Sankar Nath, Therese Anne Limbaña, Vignesh Murugan, Jian Garcia, Sanjana Singareddy

**Affiliations:** 1 Psychiatry, BronxCare Health System, Bronx, USA; 2 Surgical Oncology, Tata Medical Center, Kolkata, IND; 3 Research, California Institute of Behavioral Neurosciences and Psychology, Fairfield, USA; 4 Dermatology, College of Osteopathic Medicine, New York Institute of Technology, Old Westbury, USA; 5 Internal Medicine, California Institute of Behavioral Neurosciences and Psychology, Fairfield, USA; 6 Internal Medicine, Bhaskar Medical College, Hyderabad, IND

**Keywords:** elderly, mathadone maintenance, methadone, methadone maintenance treatment, mortality, opioid

## Abstract

Opioid dependence is a serious public health concern, particularly for older individuals who have a high prevalence of comorbid conditions. To effectively manage opioid use disorder (OUD), methadone maintenance treatment (MMT) is crucial; however, the MMT poses certain challenges for the aging population. The purpose of this review is to evaluate the impact of MMT on health outcomes, identify predictive factors for mortality, and assess mortality rates among older individuals receiving MMT.

A systematic search was performed across databases, including PubMed, Scopus, Web of Science, and Google Scholar, adhering to the Preferred Reporting Items for Systematic reviews and Meta-Analyses (PRISMA) guidelines. Studies included were published between January 2000 and December 2023, focused on elderly patients (60 years of age and older) receiving MMT and provided information on death rates. A total of 15 studies were examined. The main causes of death for older MMT patients were overdose, respiratory issues, and cardiovascular diseases. The annual mortality rates for these patients ranged from 2% to 10%. Treatment outcomes and mortality were significantly impacted by comorbid conditions. Greater treatment adherence and longer care periods were observed in older individuals, which correlated with better health outcomes and lower mortality.

This review makes clear how elderly MMT patients with addiction and chronic health issues require integrated care models. Treatment effectiveness may be further increased by gender-specific interventions. For this aging population, policy reforms and enhanced healthcare support are essential. To enhance clinical results and lower mortality rates among older individuals enrolled in MMT programs, comprehensive age-appropriate care models are crucial. Long-term health outcomes should be investigated further and evidence-based treatments for older individuals with OUD should be developed.

## Introduction and background

The problem of opioid dependence has become an increasingly serious public health issue, especially among older individuals who present with a high rate of comorbid illnesses. The aging of the population and greater longevity in opioid-dependent individuals has led to a rise in methadone maintenance treatment (MMT) demand among the elderly patients [1]. A study published in 2023 in *The Journal of Pain* found that about 5.5% of older adults aged 65 and over had used opioids for chronic pain, with a notable percentage at a risk of dependence [2]. Another study published in *JAMA Network Open* in 2022 estimated that approximately 8% of the older adults had been prescribed opioids in the past year, and a smaller, yet significant proportion, developed dependency issues [3]. Elderly opioid users often have a multimorbid profile, with higher rates of comorbidities such as diabetes, psychiatric disorders, and cardiovascular diseases, which can impact treatment response and increase mortality risk [4], which may have implications for treatment response and mortality [5,6].

According to more recent research, older individuals receiving MMT may fare just as well as younger patients, possibly as a result of higher levels of adherence and stabilization. Nevertheless, sufficient control is required due to the significant risk of death posed by aging and coexisting medical conditions [7]. Understanding these risks also aids in the development of time-specific treatment plans that improve care and clinical outcomes for elderly MMT patients.

Objectives

The objectives of this study are to systematically review the literature on mortality among elderly MMT patients and its associated factors. This review aims to integrate the current literature and, through this synthesis, provide a better idea of how we can apply these insights into clinical practice, as well as frame future research that may lead to delivering personalized interventions for elderly individuals with opioid use.

## Review

Overview

The use of opioids for medical or non-medical purposes can cause opioid use disorder (OUD), a chronic condition characterized by compulsive consumption and overall adverse consequences. Prescription pain relievers, heroin, and synthetic opioids, such as fentanyl, are examples of opiates that have high abuse potential. A growing epidemic of OUD has resulted in a serious problem of public health consequences for society, the economy, and healthcare. According to the 2023 National Institute on Drug Abuse (NIDA) report, approximately 2.5 million people in the United States were estimated to have an opioid use disorder, reflecting the widespread nature of the epidemic [8].

For several decades, MMT has been the foundation of opioid addiction treatment. In methadone, a synthetic opioid agonist attaches to the same receptors in the brain as other opioids; but unlike other opioids, methadone will diminish withdrawal symptoms and cravings when used properly, yet it will not produce nearly the same level of euphoria [9]. It enables patients to take part in treatment, which can ultimately contribute to the reduction or cessation of harmful behaviors linked with illicit opioid use [10]. In this regard, the historical focus of MMT has been on relatively young populations, especially individuals in their 20s and/or 30s. Nevertheless, the face of opioid addiction is evolving. The rates of drug and opioid use have been increasing since the end of World War II, particularly among those born between 1946 and 1964 (i.e., the "baby boomer" generation) as they age. This cohort is currently transitioning into older age, leading to an expanding population of elderly people with OUD [11].

Older individuals with OUD have been shown to experience several unique issues, which is supported by research. Not only that, they have multiple comorbid conditions - cardiovascular diseases, metabolic illness (such as diabetes), chronic pain from arthritis, and anxiety and depression. Comorbidities may impede treatment and prognosis, even leading to an increased risk of mortality [12]. For example, an older study suggested that patients aged 60 and over in MMT programs had more comorbid physical health problems and were often prescribed multiple medications, thus creating the potential for drug-drug interactions or polypharmacy [6].

Mortality among older individuals on MMT differs significantly. The annual mortality rates have been reported to range from 2% to 10% with the predominant causes of death attributed to cardiovascular diseases, respiratory conditions, and overdose [7,13]. This extended history of opioid use, along with the normal aging process and multiple chronic health conditions, leads to these rates.

There are also gender differences related to treatment response. For those in remission, older females on MMT demonstrate more favorable health-related outcomes relative to their male peers. Women are generally more compliant in seeking healthcare services compared to men. Such behavior is often associated with improved health and adherence to treatment. MMT can establish relationships with healthcare workers, enhance medical adherence among women, and likely promote regular follow-ups, leading to better treatment outcomes [14]. These differences might relate to their health-seeking behaviors, social support networks, or other aspects of the way they use opioids [15,16].

Dose-response is determined by differences in pharmacokinetics/pharmacodynamics related to biological sex, including methylphenidate blood levels and half-life, as well as the overall clinical response. Hormonal changes in women can alter how drugs are metabolized and the response to opioids, leading to changes in outcomes from treatment [17].

Compliance and the time it takes for methadone treatment are key to healing. Research has demonstrated that extended durations of care and adherence to MMT are connected with several better health outcomes, including lower likelihoods of all-cause-death. Indeed, older individuals may have better adherence to treatment protocols, which in part likely accounts for such outcomes [18].

Given this landscape, new integrated care models that target both addiction and other chronic health conditions as they typically present in older individuals are urgently needed. Policymakers and healthcare providers will need to create and implement strategies that account for the particularly challenging problems faced by elderly opioid users. Examples of these are modifying policies, strengthening the health care support system, and inpatient management that integrates addiction treatment with chronic disease [19-21].

Methods

This systematic review was based on the Preferred Reporting Items for Systematic Reviews and Meta-Analyses (PRISMA) guidelines [22]. For this review, we searched multiple electronic databases, including PubMed, Scopus, Web of Science, and Google Scholar. Our inclusion and exclusion criteria can be found in Table 1.

**Table 1:** Inclusion and Exclusion Criteria MMT: Methadone Maintenance Treatment

Criteria	Inclusion Criteria	Exclusion Criteria
Population	Studies involving elderly individuals (aged 60 and above) undergoing MMT.	Studies do not focus on elderly populations (aged 60 and above).
Intervention	Studies examining methadone maintenance treatment as the primary intervention for opioid dependence. Studies that may include MMT in combination with other interventions, provided the effects of MMT are reported separately.	Studies that do not include methadone maintenance treatment as a primary intervention. Studies focused solely on other forms of opioid substitution therapies without including MMT.
Outcomes	Studies reporting on mortality rates among elderly individuals on MMT. Studies identifying factors associated with mortality in this population, such as comorbid conditions, duration of treatment, and adherence.	Studies that do not report on mortality rates or factors associated with mortality. Studies that focus exclusively on other outcomes such as quality of life, drug use patterns, or psychosocial outcomes without addressing mortality.
Study Design	Cohort studies, case-control studies, randomized controlled trials, and longitudinal studies. Systematic reviews and meta-analyses provide comprehensive data on the topic.	Cross-sectional studies, editorials, commentaries, and letters to the editor. Non-peer-reviewed articles, including conference abstracts and dissertations.
Language	Studies published in English.	Studies published in languages other than English.
Publication Status	Peer-reviewed articles.	Gray literature, including reports, policy documents, and conference proceedings, unless they provide substantial and relevant data that is peer-reviewed.

We developed the following search strategy to identify the relevant studies on mortality in older MMT recipients. We searched databases with a combination of controlled vocabulary (e.g., Medical Subject Headings (MeSH) terminology) and free-text terms. Search terms, such as elderly, older individuals, seniors, and aging were used to identify the population. In the literature search, additional terms (e.g., methadone maintenance treatment (MMT)) were also used to capture relevant studies dealing with our intervention of interest-opioid substitution therapy and suboptimal adherence in oral compound folate (OCF). The outcomes were searched for using mortality, death, survival rates, and mortality rate. Search terms were combined using the Boolean operators (AND and OR). The search strings were constructed to be as sensitive as possible while including the necessary selection criteria for relevance.

Risk of bias

The studies included in the review exhibited several biases, including selection bias due to non-random sampling, measurement bias from inconsistent definitions of mortality and comorbidities, and attrition bias from high dropout rates in longitudinal studies. Confounding bias was prevalent due to inadequate control of variables like comorbidities and socioeconomic factors. Reporting bias was noted, with a focus on positive outcomes and underreporting of adverse events. Small sample sizes and geographic limitations reduced generalizability, and publication bias was present due to the exclusion of non-English and gray literature, affecting the overall reliability of the findings.

Discussion

**Figure 1:**
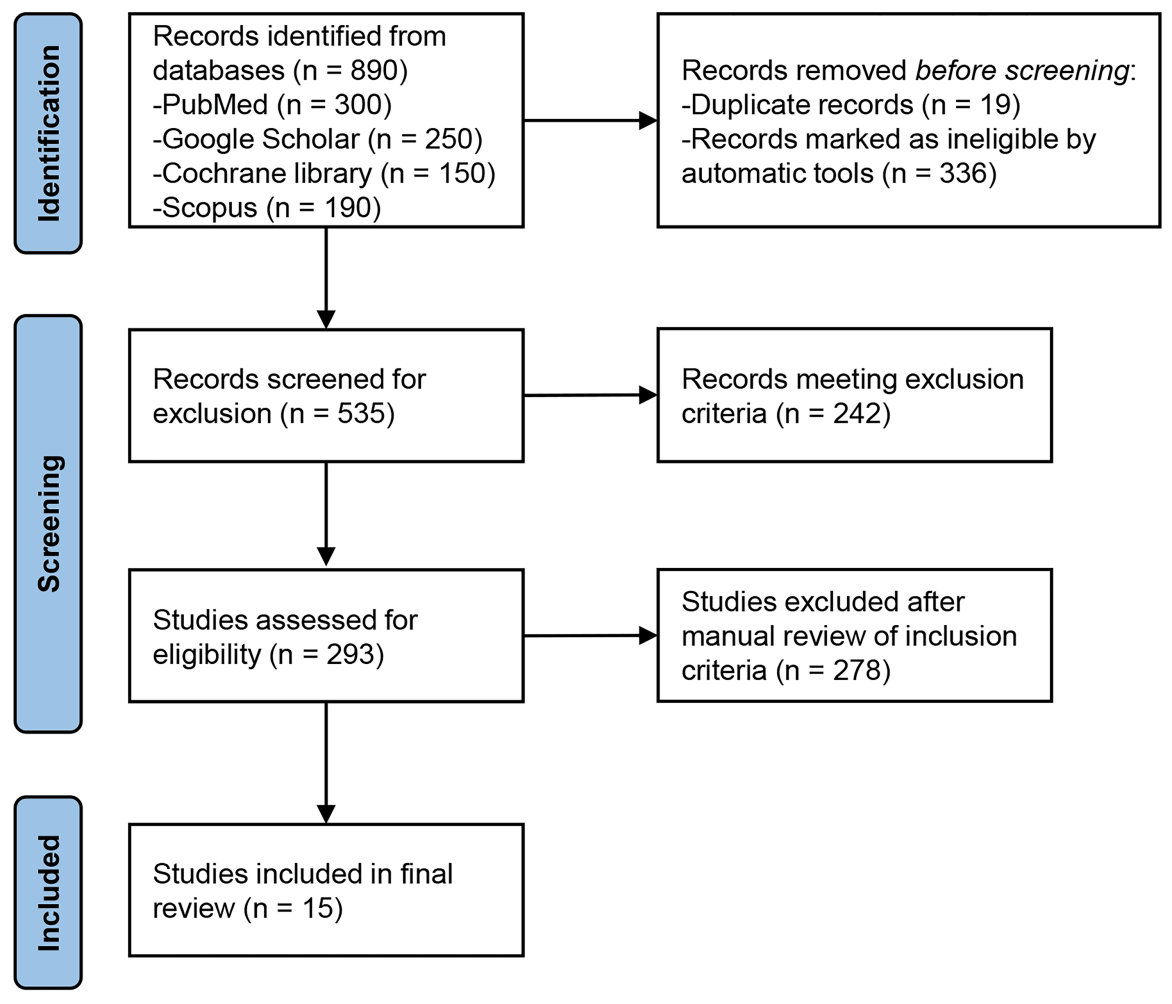
PRISMA Flowchart PRISMA: Preferred Reporting Items for Systematic reviews and Meta-analyses

Our initial search yielded 890 total records (Figure 1). We excluded 19 duplicate records and 336 ineligible records as determined by database filtering, leaving 535 for screening. From these, we excluded 242 and 293 remained. Finally, we excluded 278 after a manual review. Finally, 15 studies were available for the analysis of mortality and related risk factors for elderly patients using MMT (Table 2). The studies uniformly found a growing proportion of older individuals in MMT programs, which reflects the aging population and ongoing demand for opioid dependence treatment among elderly patients [7,23]. This change in demographics demands a deeper consideration of the specific challenges and requirements within these programs.

**Table 2:** Summary of Included Studies on Mortality in the Elderly Patients Utilizing Methadone Maintenance Treatment MMT: Methadone Maintenance Treatment

Study	Study Period	Location	Design/Method	Population (Sample Size)	Age Range	Key Features	Major Findings on Aging	Major Findings on Opioids	Recommendations
Han et al. 2019 [23]	1996-2012	New York, USA	Secondary analysis of administrative data	Individuals in opioid treatment programs (N=50,000)	Above 60 years	Examined age trends, gender differences, primary substances used	The proportion of elderly (60+) increased, and more females in the older group	Heroin primary substance, small increases in alcohol and marijuana use	For more research on elderly opioid users, new care models needed
Grella and Lovinger, 2011 [16]	1978-2009	California, USA	Longitudinal follow-up of methadone patients	Original sample N=914, followed-up N=450	Above 60 years	Identified heroin use trajectories, gender differences in remission	Women are more likely to enter remission quickly	High rates of persistent drug use over 30 years	Treatment should address long-term drug use patterns and gender differences
Lofwall et al. 2005 [6]	Not given	Baltimore, USA	Cross-sectional survey	Older opioid maintenance patients (N=67)	Above 60 years	Examined physical and mental health characteristics, urine toxicology	Older patients had poorer physical health, more prescribed medications	The older group had fewer positive urine screenings for opiates	Develop more effective healthcare strategies for elderly opioid users
Rosen et al. 2011 [24]	1990-2009	United States	Literature review	Studies involving older heroin users and methadone patients (N=varied)	Above 60 years	Synthesized findings on aging heroin users	Highlighted the need for more research on older individuals in MMT	Findings inconsistent, need for larger sample sizes	Coordinated efforts needed for research and care for aging opioid users
Hser et al. 2017 [7]	1974-1997	California, USA	Longitudinal study	Original N=581, followed-up N=242	Above 60 years	Followed heroin addicts for over 30 years	Identified persistent drug use into the late 60s	Early intervention critical for sustained recovery	Strategies should include stress coping, early cessation mechanisms
Fareed et al. 2009 [15]	2002-2007	Atlanta, USA	Retrospective chart review	Methadone maintenance patients (N=91)	Above 60 years	Examined treatment history, comorbid alcohol misuse	Older individuals had longer treatment periods and less current heroin use	Reduction in drug use, psychiatric, medical, and legal problems	Target lifestyle risk factors and comorbid conditions for intervention
Rajaratnam et al. 2009 [25]	Not given	New York, USA	Random selection using stratified sampling	Methadone treatment patients (N=156)	Above 60 years	Examined treatment duration, history of alcohol misuse	Older individuals more likely to have longer treatment periods	Less current heroin use, overall drug use reduced	Better preparedness for methadone maintenance services needed
Moy et al. 2011 [26]	Publications up to January 2007	Not applicable	Systematic review	Individuals over 50 years with any substance disorder (N=varied)	Above 60 years	Evaluated treatment responses among older individuals	Encouraging treatment responses among older individuals	Lack of studies on illicit drug use treatments	Need for increased research and development of health services for older individuals
Hser et al. 2001 [27]	1974-1997	California, USA	Longitudinal study	Original N=581 male heroin addicts	Above 60 years	Followed heroin addicts for over 30 years	Identified groups with distinct heroin use trajectories	Persistent heroin use into the late 50s	Early intervention and long-term management strategies required
Firoz and Carlson 2004 [28]	1995-2000	Midwest, USA	Prospective cohort study	Methadone maintenance patients (N=759)	Above 60 years	Examined medical and psychiatric problems, employment	The older group had improved outcomes on drug use measures	Similar medical and psychiatric problems as younger patients	Methadone programs should consider the unique needs of older patients
Doukas 2014 [29]	2000-2014	Various	Literature review	Older individuals prescribed methadone (N=varied)	Above 60 years	Reviewed lifespan from opiate initiation to MMT	Increase in comorbid conditions with age	Chronic pain and psychiatric issues prevalent	Need for comprehensive care models tailored to the elderly
Fahmy et al. 2012 [30]	2007-2010	United Kingdom	Cross-sectional survey	Individuals aged 60+ using illicit drugs (N=500)	Above 60 years	Prevalence of drug use in older individuals	Higher rates of prescription drug misuse	Opioids and benzodiazepines are frequently misused	Public health interventions needed for older individuals
Outlaw et al. 2012 [31]	2000-2010	Various	Systematic review	Older individuals with substance problems (N=varied)	Above 60 years	Treatment outcomes for older individuals	Better outcomes in older individuals compared to younger ones	Consistent with reduced drug use and improved health	Age-specific treatment protocols recommended
Teesson et al. 2015 [13]	2001-2012	Australia	Long-term cohort study	Heroin-dependent individuals (N=615)	No specific cut-off for "older"	Mortality and remission rates	High mortality among older heroin users	Long-term follow-up critical for intervention success	Integration of mental health and addiction services
Pirona et al. 2015 [32]	2000-2015	Europe	Policy review	Aging opioid users in treatment (N=varied)	Above 60 years	Challenges for treatment systems	An increasing number of older opioid users	Need for policy adjustments to meet aging population needs	Enhanced support systems and funding required

Han et al. (2019) examined the New York administrative data through a secondary analysis of individuals receiving treatment in opioid programs [23]. There was also a greater proportion of older (60+ years) patients and females in the older age group seen with COVID-19 compared to those treated with seasonal flu. The main substances used were heroin and minor increases in alcohol and marijuana consumption. This highlights the importance of initiating novel patient-centered care depending on their age, so that elderly opioid users, in particular, can be served​.

Grella and Lovinger (2011) tracked methadone patients in California for 30 years [16]. They found that older women were also more likely to go into remission faster than their male counterparts. Women enrolled in MMT programs are more likely than men in the same methadone maintenance program to achieve remission and improved health. Potential reasons were those women possibly undertake health-seeking measures more proactively with better engagement in treatment services. This finding could point to the necessity of gender-specific interventions when treating older women. Over the 30 years, we noticed high rates of persistent drug use hinting towards the requirement of more long-term treatment strategies to manage ongoing substances​ [16].

Lofwall et al. (2005) published a cross-sectional survey of elderly patients maintained on opioids in Baltimore [6]. Compared with younger patients, older ones experienced worse physical health and were on more medications. The older group also had significantly fewer positive urine screens for opiates, reflecting greater treatment fidelity. The study underscores the urgent need for targeted healthcare systems and interventions designed to specially address these various physical health issues among older opioid users​​.

Rosen et al. (2011) performed a systematic literature review concerning elderly heroin users and methadone patients in the USA [24]. The review also emphasized that there are inconsistent results in research about older individuals on MMT, and more studies should be done by the researchers. Coordinated research and care for aging opioid users were sought.

Hser et al. (2017) conducted a 30-year follow-up of heroin addicts in California [7]. They observed consistent drug abuse up to the 60s as well as emphasized the importance of early intervention for continued recovery. Strategies identified by the study, such as stress coping mechanisms and early cessation interventions, are recommended to improve long-term outcomes.

Fareed et al. (2009) in a retrospective chart review of methadone maintenance patients in Atlanta showed that older individuals were significantly less likely to be current heroin users and more likely to have had a longer duration of treatment [15]. Older patients experienced decreases in drug use, psychiatric diagnosis, and medical and legal issues, which highlight the need to address lifestyle risk factors and comorbid conditions when planning treatment outcomes.

Rajaratnam et al. (2009) examined methadone treatment patients recruited in New York employing a stratified sampling technique [25]. They also discovered that older individuals were more likely to have a longer duration of treatment and less current heroin use. These findings accentuate the need for better preparedness regarding methadone maintenance services suited to older individuals. The findings from the study showed that older age was associated with significantly longer treatment durations. This may indicate that older patients are more likely to continue with treatment due to a greater desire for better health, or because methadone is associated with an improvement in their social and family life.

Moy et al. (2011) conducted a systematic review of treatment responses in older individuals with substance disorders [26]. Consequently, this review identified one study that examined illicit drug use treatment in older individuals and several studies providing moderate support for treatment responses among aging individuals. The researchers concluded that there should be a higher scope of clinical research and developmental health care for senior citizens

Hser et al. (2001) followed a cohort of heroin addicts in California for 33 years [27]. They saw identifiable trajectories of heroin use, the longest extending into the late 60s and with persistent heroin use. It is reinforced in this review and notes the importance of early identification along with long-term treatment to better results for older heroin users.

Firoz and Carlson (2004) published a study that also showed that older methadone maintenance patients had significantly better outcomes about drug use measures compared to younger ones [28]. At nine months following treatment admission, 61 percent of the older individuals (60 and over) had no positive urine drug screens as compared to only 35 percent in the younger group. This suggests greater adherence to treatments in older individuals. Medical and psychiatric comorbidities were not significantly different in older compared with younger patients who had similar medical and psychiatric problems. This means that older people are more compliant with MMT but they have a lot of health problems, which must be addressed in treatment programming.

Doukas (2014) conducted a descriptive literature review of older individuals on methadone [29]. In this review, comorbid conditions increased with age and there was a high prevalence of chronic pain related to cardiovascular/lung disease risk factors in the majority of studies examined as well as details on psych issues. Doukas suggested that these problems are effectively addressed by comprehensive care models adapted for elderly patients.

Fahmy et al. (2012) conducted a cross-sectional survey of drug-taking behavior among individuals aged 60 years or above in the United Kingdom [30]. For older individuals, the rates of prescription drug misuse were especially high, particularly for opioids and benzodiazepines. According to the study, these trends indicate that public health interventions designed for older individuals are needed.

Outlaw et al. (2012) conducted a study on treatment outcomes for older individuals who have high substance abuse [31]. This study found that among non-obese and obese people, older individuals might benefit from exercise more than younger ones because of less use of drugs with age or concomitant medication usage as well as better health status. They concluded that age-based treatment protocols will lead to better outcomes among older individuals.

Teesson et al. (2015) conducted a cohort study in Australia including heroin users, over the long term [13]. They reported significant attrition and high mortality rates in older heroin users and underlined the necessity of long-term follow-up for successful intervention. The study reinforces the call for integrated mental health and addiction services to enhance outcomes.

Pirona et al. (2015) carried out a policy review focused on aging opioid users in treatment within Europe [32]. The review noted a growing population of elderly opioid users and advocated policy changes to address the needs of an aging consumer base. Better support and funding for the care of elderly opioid users are needed.

Overall, these studies showed that persistent drug use continues past the age of 60, and the number of individuals in this group using MMT is increasing. There are more women in this population; however, they are more likely to enter remission than men. Older patients were also in poorer health than younger patients, had other comorbidities, and had higher mortality rates. They were usually prescribed more medications and were more likely to have longer treatment periods than those of younger patients. The outcomes were better for this group compared to younger patients.

Limitations

Our review has several limitations. First, our study is primarily qualitative, only reporting summaries of the studies. Second, we had no hypothesis to test, nor did we use statistical analysis. Third, the studies we examined may have a risk of biases. Additionally, our study could be at risk of selection bias and or methodological errors that could have limited our findings.

## Conclusions

The present review highlights the critical need for tailored, integrated care models for older individuals receiving methadone maintenance treatment (MMT), as they often face high mortality rates due to overdose, respiratory, and cardiovascular issues. This review successfully met its objectives by identifying key predictive factors for mortality, including comorbid conditions, treatment adherence, and gender differences, which impact health outcomes. The findings underscore the importance of developing age-specific, evidence-based interventions and comprehensive care approaches that address both opioid dependence and chronic health conditions to improve clinical outcomes and reduce mortality among elderly MMT patients. Future research should focus on refining these care models and exploring personalized treatment strategies to enhance long-term health outcomes for this vulnerable population.
